# EchoTilt: An Acoustofluidic Method for the Capture and Enrichment of Nanoplastics Directed Toward Drinking Water Monitoring

**DOI:** 10.3390/mi15121487

**Published:** 2024-12-11

**Authors:** Martim Costa, Liselotte van der Geer, Miguel Joaquim, B. Hammarström, S. Tanriverdi, H. N. Joensson, M. Wiklund, A. Russom

**Affiliations:** 1 Science for Life Laboratory, Department of Protein Science, Division of Nanobiotechnology, KTH Royal Institute of Technology, 171 65 Solna, Sweden; martimc@kth.se (M.C.); lgbvdg@kth.se (L.v.d.G.); selimt@kth.se (S.T.); hakan.jonsson@scilifelab.se (H.N.J.); 2 Departamento de Bioengenharia, Instituto Superior Técnico, 1049-001 Lisboa, Portugal; miguelrtsjoaquim@gmail.com; 3 Science for Life Laboratory, Department of Applied Physics, KTH Royal Institute of Technology, 171 65 Solna, Sweden; bham@kth.se (B.H.); martin.wiklund@biox.kth.se (M.W.); 4 AIMES Center for the Advancement of Integrated Medical and Engineering Sciences at Karolinska Institutet and KTH Royal Institute of Technology, 171 65 Solna, Sweden

**Keywords:** acoustofluidics, seed particle method, silica-enhanced seed particle method, microplastics, nanoplastics, acoustic trap, microfluidic-based separation

## Abstract

Micro- and nanoplastics have become increasingly relevant as contaminants to be monitored due to their potential health effects and environmental impact. Nanoplastics, in particular, have been shown to be difficult to detect in drinking water, requiring new capture technologies. In this work, we applied the acoustofluidic seed particle method to capture nanoplastics in an optimized, tilted grid of silica clusters even at the high flow rate of 5 mL/min. Moreover, we achieved, using this technique, the enrichment of nanoparticles ranging from 500 nm to 25 nm as a first in the field. We employed fluorescence to observe the enrichment profiles according to size, using a washing buffer flow at 0.5 mL/min, highlighting the size-dependent nature of the silica seed particle release of various sizes of nanoparticles. These results highlight the versatility of acoustic trapping for a wide range of nanoplastic particles and allow further study into the complex dynamics of the seed particle method at these size ranges. Moreover, with reproducible size-dependent washing curves, we provide a new window into the rate of nanoplastic escape in high-capacity acoustic traps, relevant to both environmental and biomedical applications.

## 1. Introduction

Plastics have become a staple material in human activity since their inception 70 years ago [1]. While their production and applications have developed tremendously, concerns are being raised regarding their adequate disposal and end-of-life-cycle outcomes [2]. The benefits of this material are several—cost-effectiveness, ease of production, versatility and long shelf-life. These are essential for the packaging, medical and textile industries, to name a few [3]. However, it is their resistance to biodegradability that is at the core of an increasing risk to environmental and human health as plastics accumulate in various ecosystems, from aquatic [4] to terrestrial [5]. Ultimately, this leads to the presence of micro- and nanoplastics in consumer goods like food [6] and drinking water [7]. These can be primary, produced by human activity with the explicit aim of being used as micro- and nanoparticles, or secondary, which leach from larger polymer bodies when exposed to ultraviolet radiation or abrasion [8]. In terms of size, microplastics are commonly defined as being smaller than 5 mm and larger than 1 µm, whereas nanoplastics are below that threshold. At the micro and nano size ranges, modern sample management and analytical tools struggle to provide a reliable, simple-to-answer system for micro- and nanoplastic monitoring [9]. There is a need to better understand the presence, types and life cycles of micro- and nanoplastics, considering that they have already been found in many organs of the human body [10,11,12].

Microfluidics is a field of study that has been used in the past to address accurate manipulation of micro- and nanoparticles. It has been essential for the further development of the biomedical field, bringing together disciplines such as chip manufacturing, electrical engineering, technical design and fluid mechanics directed toward biomedical solutions that aim to improve human health. These solutions are as varied as the multidisciplinary nature of the field, having been used, for example, in cancer screening [13], DNA amplification for sepsis diagnostics [14] and blood component separation [15]. While microparticles have been shown to be manipulated by many techniques [16,17], nanoparticles have been shown to be harder to manage using the same approaches [18]. The potential applications of their accurate enrichment and separation are of enormous interest for the biomedical field. (e.g., for exosome enrichment [19] or virus isolation [20]). The same can be said for the environmental area, considering the negative implications of nanoplastic contaminants in drinking water.

Acoustofluidics integrates ultrasonic sound waves with microfluidics, employing piezoelectric transducers to generate acoustic fields within microchannels. These fields allow particles to be precisely manipulated for several purposes. Examples of these are microparticle focusing [21], separation [22,23] and nanoparticle capture [24]. However, these capabilities have not been developed with sufficient throughput for high-volume environmental applications. Techniques like electrophoresis, field-flow fractionation or deterministic lateral displacement have been able to manage nanoparticles at flow rates above a few µL/min [18]. In order for new monitoring solutions to be effective for micro- and nanoplastics, it is necessary that they are able to sustain adequate performance even at high flow rates while extracting a sufficient amount for endpoint analysis. For this reason, a technique that combines high flow rates with nanoplastic capture is of great interest as a proof of concept for a future environmental monitoring applications that can capture not only microplastics but nanoplastics as well.

Previously, we developed the EchoGrid, a high-throughput acoustofluidic device for microplastic enrichment [25], which was a crucial first step toward applying acoustofluidics to drinking water applications. We employed silica as stable seed particles for microplastics to be enriched at high flow rates. These anchor points leveraged two crucial forces in acoustofluidics, the primary radiation force (Equation (1)—PRF) and the secondary radiation force (Equation (2)—SRF).

(1) $$F_{PRF}=-\left(\frac{\pi \rho_{0}^{2}a^{3}\beta_{W}}{2\lambda }\right)\cdot \left(\left(\frac{{5\rho }_{c}-2\rho_{w}}{{2\rho }_{c}+\rho_{w}}\right)-\frac{\beta_{C}}{\beta_{W}}\right)\cdot \sin\left(2kx\right)$$

where *p*_0_ is the acoustic pressure amplitude, *a* is the radius of the particle, *β_C_* is the particle compressibility and *ρ_c_* is the particle density. In the acoustic contrast factor term, there is the density of the medium *ρ_W_* and the compressibility of the medium *β_W_*. Finally, the wave number *k* can be written as 2π/*λ*, while *x* is the distance from a pressure node.

(2) $$F_{SRF}=-\frac{6\pi a_{p}^{3}a_{s}^{3}E_{ac}f_{2p}f_{2s}}{d^{4}}$$

The new terms in Equation (2) [26], for the SRF of particles close to the pressure node, are *a_p_*, the seed particle size; *a_s_*, the target particle size; *E_ac_*, the acoustic energy density of the standing wave; *f*_2*p*_, the scattering dipole coefficient of the seed particle; *f*_2*s*_, the scattering dipole coefficient of the target particle; and *d*, the interparticle distance.

The PRF depends primarily on the particle size (*a*^3^) for its magnitude, whereas the SRF also depends on interparticle distance (*a_p_*^3^*a_s_*^3^/*d*^4^). As the size of particles in a system increases, both the PRF and the SRF increase, although the center-to-center distance between particles becomes more pronounced (*d*), which must also be considered. The PRF drives particle motion toward the acoustic nodes and antinodes, because of the scattering of the acoustic wave on the particle, whereas the SRF is felt between particles as the acoustic field scatters between them. We intend to highlight how the PRF and the SRF act synergistically, wherein larger clusters attract more particles (PRF), which keep each other together with interparticle forces (SRFs), allowing a further increase in cluster size (PRF).

This distinction is essential when considering systems handling high concentrations of particles. In the silica-enhanced seed particle grid, a large number of highly compact silica beads are kept in place by the wave itself (PRF) and by the scattering occurring in between the many particles in the cluster (SRF). As micro- or nanoparticles flow through the channel, the PRF remains stable (it considers each cluster as a single large particle), whereas the SRF continues to scale with each particle added. This means that dense pre-seeded acoustic traps, with their initial high concentration of particles, develop a powerful combination of PRF and SRF to trap incoming particles, even at high flow rate, and even at low nanoparticle size ranges.

In this paper, we report the EchoTilt, an acoustofluidic method designed to maximize nanoplastic capture in an array of acoustic nodes using the silica-enhanced seed particle (S-ESP) method, building off our previous work [25]. For this, we employed a computational approach for optimizing the theoretical ideal angle that predicts the most uniform nanoparticle–cluster interaction. We demonstrate for the first time that it is possible to capture nanoparticles down to 25 nm and enrich them even at the high flow rate of 5 mL/min. Our comprehensive study quantifies, through fluorescence, the enrichment rates of 500, 200, 100, 50 and 25 nm microplastics in silica clusters, highlighting the impact of size on the behavior of these particles. Finally, we performed washing experiments to understand the size-dependence of nanoparticle leaching from silica seed particles, paving the way for a better understanding of high-capacity seed particle systems for environmental or biomedical applications.

## 2. Materials and Methods

### 2.1. Acoustofluidic Device

#### 2.1.1. Fabrication and Modeling

**Figure 1:**
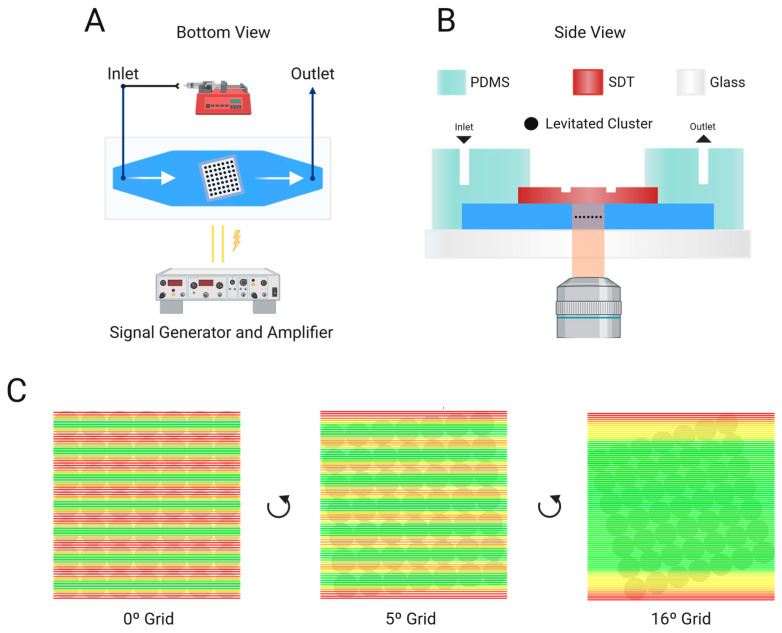
(**A**) Bottom view of the EchoTilt microchannel, with a connected syringe pump and signal generator. (**B**) Side view schematic of the microfluidic assembly. (**C**) Schematic of the algorithm angle sweep (showing 0, 5 and 16 degrees as examples), with highlighted flow lines crossing the tilted grid. Green lines signify a high score of flow lines–cluster interaction, yellow lines a medium score and red lines a low score.

The acoustofluidic device (Figure 1A,B) was made by integrating a piezoelectric transducer with a polydimethylsiloxane (PDMS) microchannel sealed with a glass reflector layer. The surface displacement transducer (SDT) was created by milling a 0.1 mm deep indent into the backside of a 30 × 20 mm^2^ piezoelectric transducer (Pz-26, Parker Meggitt, Coventry, UK), determining the trapping region. The tilted shape of this indentation and its milling files were designed and generated using AutoCAD and Fusion 360 (Autodesk Inc., San Francisco, CA, USA). For the milling of both the SDT and the mold, a computerized numerical control (CNC) milling machine (Modela MDX-40A 3D Milling Machine, Roland DGA, Irvine, CA, USA) was used.

The transducer was then integrated with the PDMS microchannel. This was done through standard soft lithography. The polymethyl methacrylate mold was milled from a 0.5 mm PMMA substrate and placed in a Petri dish. PDMS was mixed in a 10:1 ratio of monomer to curing agent (SYLGARD 184, Dow Corning, Midland, MI, USA) and was then poured into the mold, where the SDT had already been placed. This assembly was degassed for 45 min and then cured at 60 °C for 90 min. After fluidic connections were made with a 20 ga syringe (Instech Laboratories Inc., Plymouth Meeting, PA, USA), the microchannel was sealed with a glass reflector layer. This was done by using an oxygen plasma treatment to activate the surface of a 75 × 38 × 1 mm^3^ borosilicate microscope slide (Corning Inc., Corning, NY, USA) and of the PDMS slab, which were then bonded together through direct contact.

#### 2.1.2. Device Modeling

Similarly to previous work [25,27], two theoretical models were combined to predict the operation of the device. A 1D model based on an acoustic transmission line was used to determine the main axial resonances generated by the various material layers of the transducer. Then, COMSOL Multiphysics (COMSOL 6.1, COMSOL Multiphysics, Stockholm, Sweden) was used (Table S1) to find the adequate lateral dimensions of the SDT protrusion to maximize the lateral resonances of the system through an eigenmode simulation. This work allowed a sweep of various eigenmodes and lateral indent dimensions that informed the selection of an ideal acoustic field node distribution. The frequency that generated this acoustic field was then confirmed by an impedance sweep (Z-Check 16777k, Analog Instruments, Wilmington, MA, USA) to determine the SDT’s admittance peaks. Based on this, the frequency of 2.020 MHz was selected to provide a stable and well-defined grid.

#### 2.1.3. Trapping Area Tilt Modeling

A 7 × 7 grid square was constructed in Python based on COMSOL simulations (Figure S1 and Table S1), generating an ideal resonance mode for a 10 mm wide square SDT. This grid was then overlaid with equidistant lines to represent laminar flow, where nanoparticles were assumed to follow fluid lines.

Subsequently, a scoring system was developed based on the acoustic pressure decay function obtained from further COMSOL Multiphysics simulations. This function was then normalized to a scoring range of 0 to 1. In this scoring system, a score of 1 indicates that the flow line passes through the center of the cluster, while a score of 0 signifies that the flow line falls outside the effective area of the cluster’s influence. The radius of each cluster was delineated to be 0.49 arbitrary units (a.u.).

The developed algorithm (Figure 1C) then employed an angle sweep between 0 and 90 degrees, allowing the scoring system to evaluate the interaction between fluid flow lines and the acoustic field for every SDT orientation. Each flow line was assigned a score based on its interaction with the clusters. To determine the overall effectiveness of the acoustic field at each tilt angle, a final score was computed by taking the median value of all the individual scores. Based on these scores, the optimal flow line interaction with the acoustic cluster field was observed in the Python script at a tilt angle of 16°, which we then fabricated. While the principle behind this model suggests an improvement, we have not validated it experimentally, as it was not possible to fabricate devices with various angles for comparison.

### 2.2. Experiment

#### 2.2.1. Setup and Samples

The experimental setup used with the EchoTilt was composed of a signal generator (DS345, Stanford Research Systems, Sunnyvale, CA, USA), a 4× current amplifier (ADA4870ARR-EBZ, Analog Devices, Wilmington, MA, USA), a syringe pump (PHD Ultra, Harvard Apparatus, Holliston, MA, USA) and a fluorescence microscope (Axiovert 135M, Carl Zeiss AG, Oberkochen, Germany). In terms of fluorescence acquisition, the exposure time used was 200 ms at a magnification of 10×. The fluidic connections were made with plastic tubing (BTPE-60, Instech Laboratories Inc., Plymouth Meeting, PA, USA), metallic connectors (SC20/15, Prime Bioscience, Singapore) and plastic syringes (Plastipak, BD Bioscience, Woodbridge, NJ, USA).

The nanoparticle suspensions were created by diluting a stock of polystyrene nanoparticles. The ratio used was 1 mL/1000 mL (stock/MQ water, 1000× dilution). This method was followed for every particle size, including 500 nm (Red Fluorescent Fluoro-Max, ThermoFisher, San Jose, CA, USA), 200 nm (Green Fluorescent Fluoro-Max, ThermoFisher, San Jose, CA, USA), 100 nm (Green Fluorescent Fluoro-Max, ThermoFisher, San Jose, CA, USA), 50 nm (Green Fluorescent Fluoro-Max, ThermoFisher, San Jose, CA, USA) and 25 nm (Green Fluorescent Fluoro-Max, ThermoFisher, San Jose, CA, USA), and a vortexer was used to prevent sedimentation from occurring. The silica solution was created by mixing 1 wt% detergent (Tween-20) with 10 µm silica particles (Sigma-Aldrich, Buchs, Switzerland) in deionized water. To prime the chip, deionized water was used. To clean the chip between experiments, 50% ethanol was used.

#### 2.2.2. Experimental Procedure

This work relied on the silica-enhanced seed particle method. For this, a high-concentration silica solution is flowed through the channel, and then the acoustic field is activated. This creates the grid of silica clusters, which is then washed with MQ water at 15 mL/min to remove the excess silica from the device. Finally, the nanoplastic sample is loaded into the device at the flow rate relevant to the experiment, which results in the capture of the nanoparticles within the silica clusters. An image was taken at every time point with the fluorescence microscope. These fluorescence data were processed using ImageJ 1.53a, and a distinct color was attributed to each particle type. Furthermore, all graphs were created using GraphPad Prism 10.4.0 (GraphPad Software Inc., La Jolla, CA, USA).

## 3. Results and Discussion

### 3.1. Flow Simulation and Tilting

We developed the EchoTilt method to increase the interaction between flow lines and levitated clusters in a grid of acoustic nodes, for nanoparticle enrichment. The device is based on previous work [25], where a straight grid was used to enrich microplastics at a high flow rate. The crucial alteration between the two designs is the tilt angle of the milled slot on the back of the SDT. We used a combination of COMSOL and Python 3.10 modeling to determine the theoretical ideal tilt of a 7 × 7 grid of acoustic nodes that would have the most uniform interaction ‘score’ across the height of the trapping area.

**Figure 2:**
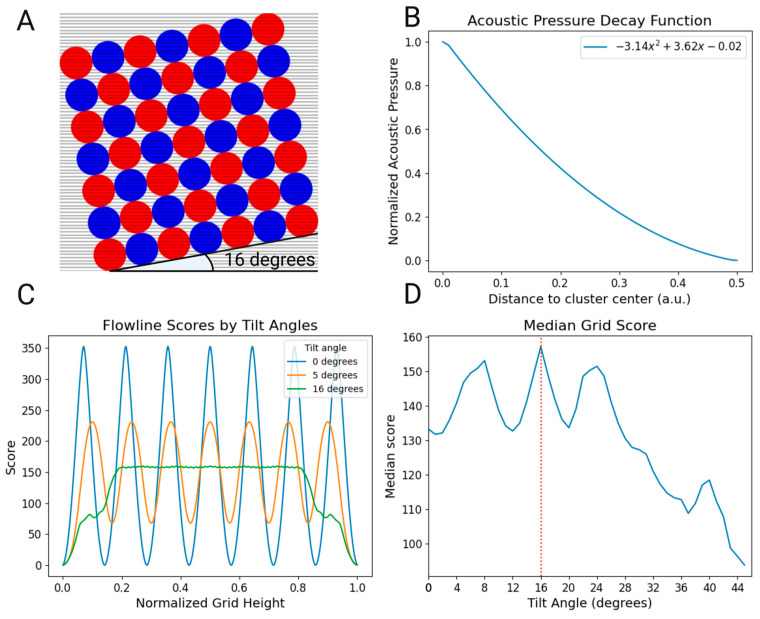
(**A**) Sixteen-degree-tilted acoustic field overlaid with a visual representation of the intersecting flow lines. (**B**) Acoustic pressure decay function extracted from COMSOL Multiphysics normalized for a 0–1 score. (**C**) Depiction of flow line score variation across the active capture area for different tilt angles in blue, orange and green (0, 5 and 16 degrees, respectively). The x-axis represents the normalized height, where 0.0 corresponds to the beginning and 1.0 to the end of the cluster array. The y-axis represents the score value associated with a flow line at each particular height. (**D**) Graph of the median grid score obtained by the algorithm, with the maximum median score at the 16° tilt angle highlighted.

The eigenfrequencies of the SDT were simulated using COMSOL Multiphysics. Of these, the frequency of 2.020 MHz was selected, which corresponded to a 7 × 7 array of acoustic nodes. Using the COMSOL Multiphysics line measurement tool (Figure S1A), we extracted the plot of the acoustic pressure variation with the distance from the cluster center, and normalized it to a 0–1 range (Figure 2B). This normalized function was inserted into a Python scoring algorithm, which swept the tilting degrees from 0 to 45°, and collected the cumulative score of the flow line–cluster contact (Figure 1C).

In Figure 2, we detail the results from this theoretical study. The schematic of the flow line–cluster interaction can be found in Figure 2A, and the flow line scores along the height of the grid, per angle, are seen in Figure 2C. Here, we observe that we have maximum variability for a 0-degree grid. The seven peaks correspond to the seven clusters in the vertical direction of the grid and show the discrepancy between the flow lines crossing through the center of each cluster (as they are perfectly aligned), and the flow lines that are in between each cluster, at the maximum distance. In the theoretically ideal 16-degree angle, we achieve stability of interaction, particularly between 0.2 and 0.8 of the grid height, with only the edge of the channel showing less interaction (as there is no capture area there). This central uniformity of flow line–cluster interaction suggests that a representative sample of the flowing nanoparticles is forced to interact with the levitated clusters, instead of having flow lines, as in the case of 0 degrees and 5 degrees, that escape in the maximum distance between clusters.

This normalized function was then applied to every flow line during a sweep of each tilt angle, and the total median score was collected for each (Figure 2D).

Furthermore, we used the total median score of the grid to select the ideal tilt angle. We selected the median instead of the mean (Figure 2D) as a measure of central tendency due to its robustness against extreme values. In other words, the median expresses the typical interaction between flow lines and clusters, as the central tendency is more relevant than the aggregate intensity. This makes it more suitable for comparing different grid configurations, as is the case with our angle sweep. Based on this theoretical work, we selected the 16-degree angle as the one to be fabricated, with its outer edge lower values being less relevant than the central area. Moreover, nanoparticles that escape through the sides are also brought to the center area by the acoustic streaming within the microchannel.

**Figure 3:**
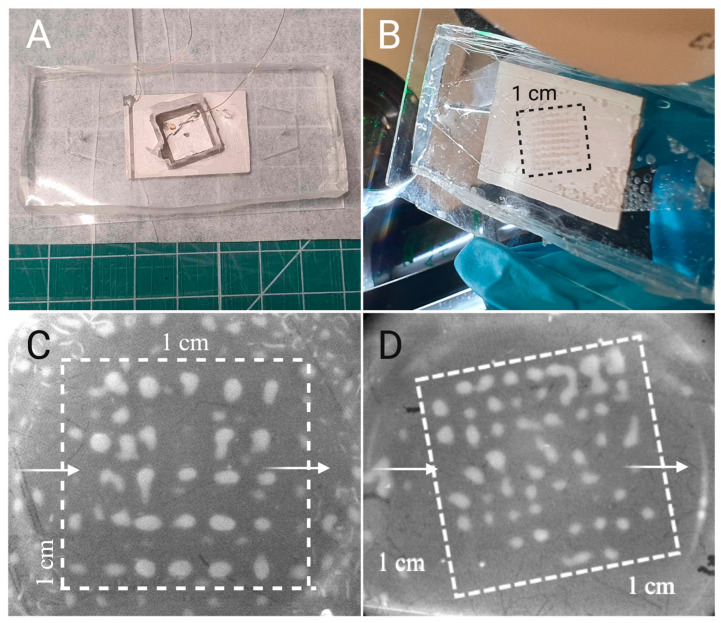
(**A**) Picture of the EchoTilt device from the top, showing the milled slot in the back of the SDT. (**B**) Picture of the bottom (through the glass) of the EchoTilt device with the highlighted 1 cm wide silica cluster grid, visible with the naked eye. (**C**) Original EchoGrid (0-degree tilt) active trapping area with well-defined clusters. The surrounding silica outside of the square is also patterned with weaker forces and is washed off before the experiment. (**D**) EchoTilt optimization, showing the 16° tilted trapping area and the organized clusters within it. In (**C**,**D**), the flow direction is from left to right.

We milled the shape of the desired tilted capture area in the back of the SDT (Figure 3A), defining the trapping area in the microchannel (Figure 3B). By tilting the straight grid (Figure 3C) by 16 degrees (Figure 3D), we eliminate the straight empty lines in between the silica clusters (red lines in Figure 1C). Note that there is less silica outside of the trapping area in Figure 3D. This is because this picture was taken after the washing step detailed in the Materials and Methods Section. The clusters can be tuned to be more numerous and wider by using a larger initial silica concentration, also depending on handler proficiency.

### 3.2. Nanoplastic Enrichment

The enrichment of nanoparticles has been a challenge in microfluidic separation in general, particularly at high flow rates. In acoustofluidics, the main techniques used for manipulating particles at the nanoscale are acoustic streaming [28] and the seed particle method [29]. While the former relates to vortices in the fluid layer that carry nanoparticles, the latter uses larger seed particles to create interstitial anchoring points where the analytes can be trapped. At this time, the minimum trapped nanoparticle size using this method has been reported to be 100 nm, at a flow rate of up to 10 µL/min [29]. The maximum flow rate, pertaining to enriching 270 nm beads, has been reported to be 30–200 µL/min using silica seed particles. These were used because of their higher dipole scattering coefficient and retention coefficient of material, among other advantages [24]. Silica particles are also attractive for drinking water monitoring applications owing to their distinct chemical fingerprint when combined with endpoint analysis, such as spectroscopy, allowing for the detection of enriched samples without interference from the seed particles in which they are captured.

In this work, we further leverage the S-ESP to investigate the minimum particle size that can be captured, as well as the maximum flow rate of solution from which enrichment is possible. To evaluate whether the EchoTilt method can achieve better outcomes in terms of minimum particle size and throughput, we flowed five different fluorescent particles (500 nm, 200 nm, 100 nm, 50 nm and 25 nm) through an array of silica seed particle clusters at two distinct flow rates (5 mL/min and 2 mL/min) and evaluated their enrichment measured over an arbitrary cluster.

#### 3.2.1. Enrichment of Larger Nanoplastics (500, 200 and 100 nm)

Capturing nanoparticles using acoustic trapping is known to become more challenging as the target analyte becomes smaller [25]. For this reason, this study first evaluated whether high-throughput enrichment of these larger nanoparticles was possible, even though the primary radiation force (Equation (1)—PRF) is weaker, considering how powerfully it decays with a reduction in particle size. This suggests that the secondary radiation force (Equation (2)—SRF) has a more relevant role in trapping nanoparticles when compared to microparticles, especially when considering high concentrations of seed particles.

**Figure 4:**
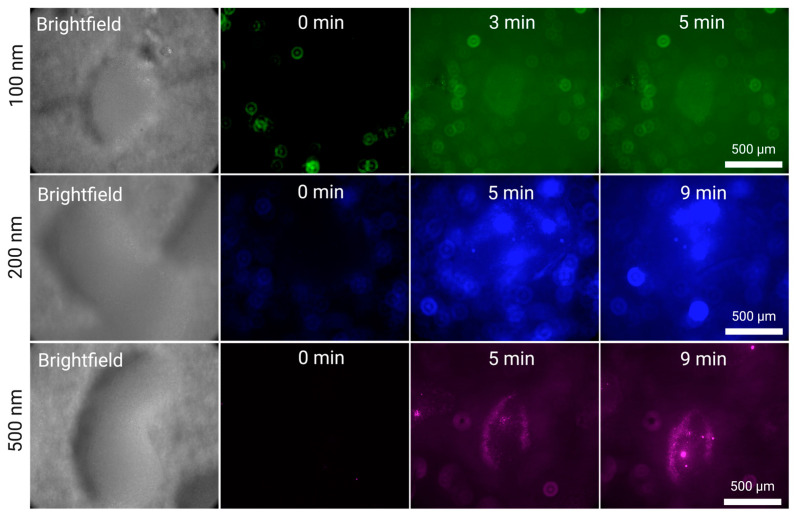
Silica-enhanced seed particle method nanoparticle enrichment at a flow rate of 5 mL/min using the EchoTilt device. The increase in fluorescence is attributed to the flowing nanoparticle solution interacting with the cluster. The particles used were 100 nm (green), 200 nm (blue) and 500 nm (magenta).

In Figure 4, we present the increase in fluorescence across time in a silica cluster hovering above the surface of the transducer. It is possible to see in some images colored background light. This is due to a combination of solution fluorescence and light reflection from the surface of the transducer.

We observe clear large nanoparticle particles (100, 200 and 500 nm) for all experiments, for both flow rates (5 mL/min and 2 mL/min). At the high flow rate (5 mL/min), some clusters can slightly drift from their initial position until they settle in an equilibrium state caught between the acoustic field and the Stokes drag of the fluid.

In the case of 500 and 200 nm, we note the formation of nanoparticle kernels in the interstitia of the silica cluster, which increase in fluorescence as the experiment continues, suggesting that their increasing size continues to speed up the collection of more nanoparticles, a similar phenomenon to what we observed with microparticles [25]. This is particularly visible for t = 3 min and t = 5 min for 200 nm particles. When compared to the 500 nm case, we observe that the larger the silica cluster, the more it facilitates the formation of these kernels. This is because large silica clusters act as a porous funnel where flowing nanoparticles have more potential anchoring positions. This translates to more kernels during enrichment, which, as they grow, become more susceptible to the PRF in addition to the SRF.

The high-capacity grid of silica clusters highlights two important observations: first, larger silica particles translate into a larger area of capture, simultaneously allowing higher throughput without excessive sample loss, and they facilitate the formation of nanoparticle kernels within them, with act as better attractors of nanoparticles and immobilize them more firmly, increasing their relevance for endpoint analytical applications.

In the lower flow rate experiment (2 mL/min), Figure S2, we also observe a smaller 100 nm kernel, even though it is a rare occurrence compared to the larger particles. The lower flow rate also makes it clear that the general fluorescence of the cluster increases first at the edges of the silica seed particle, and advances toward the center (particularly in 200 nm—Figure S2), with kernels being the exception to this phenomenon.

The graphs pertaining to the fluorescence progression of these experiments can be found, commented, in Section 3.2.3.

#### 3.2.2. Enrichment of Smaller Nanoplastics (50 and 25 nm)

For smaller nanoparticles (50 and 25 nm), we also used flow rates of 5 mL/min and 2 mL/min. In these experiments, we could not observe any formation of large fluorescent kernels. Nevertheless, to our knowledge, 25 nm plastics have not been captured using acoustofluidics.

**Figure 5:**
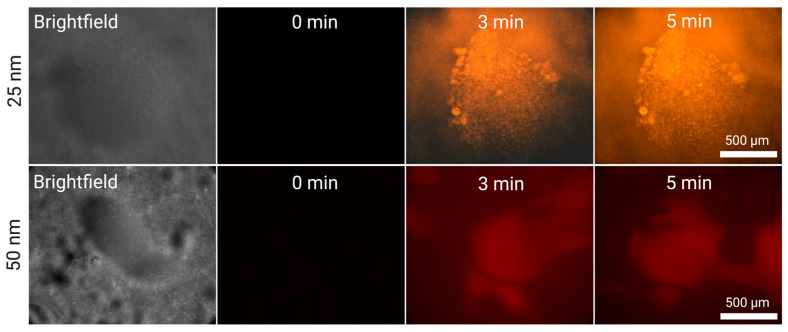
Silica-enhanced seed particle method nanoparticle enrichment at a flow rate of 5 mL/min using the EchoTilt device. The increase in fluorescence is attributed to the flowing nanoparticle solution interacting with the cluster. The particles used were 25 nm (red), and 50 nm (orange).

In Figure 5, we observe the growing fluorescence of the silica clusters during nanoparticle enrichment (50 and 25 nm) at 5 mL/min. In both cases, we cannot observe specific particles fluorescing even at high magnifications; instead, the fluorescence is indistinguishable from the silica particles themselves. Unlike what is observed in the case of larger nanoparticles, particularly 200 and 500 nm, there is not a considerable increase in fluorescence across time. This suggests that the acoustic silica clusters saturate earlier, and the nanoparticles being captured are at the same level as, or slightly below than, those escaping. Specifically, the 25 nm particle clusters gain 14% fluorescence compared to their first normalized time point (1 min), over 5 min, and the 50 nm particle clusters lose 33% fluorescence. These values are much lower than the 128% and 159% gained by 200 and 500 nm, respectively, after 5 min.

Our results show that smaller nanoparticles (100, 50, 25 nm) are better captured at lower flow rates (2 mL/min—Figure S3) than at higher flow rates (5 mL/min—Figure 5). After 9 min, the 25 nm particles gained 31% of their initial normalized fluorescence and the 50 nm particles gained 28%. However, the increase was 87% and 135% for the 200 and 500 nm, respectively, which is lower than what was obtained at 5 mL/min. The intermediate nanoparticle, 100 nm, showed a 1% decrease (effectively saturated after the first minute) at 5 mL/min, and it increased by 36% at 2 mL/min. These values were calculated by taking the average of the measurements, and calculating the percentage increase compared to the first normalized value at t = 1 min.

These observations suggest that higher flow rates can sustain better enrichment outcomes for larger particles (200 and 500 nm) than for smaller particles (100, 50 and 25 nm). For nanoparticles where the PRF is more present, higher flow rates lead to better formation of kernels, which in turn can capture more particles. In situations where the SRF dominates (100, 50 and 25 nm), there appears to be a limit from which the flow rate can be too high for further enrichment after the baseline 1 min mark. In previous work [25], we noted that, while higher flow rates (5 mL/min) demonstrated lower absolute efficiency in capturing microplastics (10 and 23 µm), they were able to capture more particles per minute than the lower flow rates (2 mL/min). The results presented here regarding nanoparticles suggest that there is a lower limit to the trade-off between particles captured and flow rate, and that it is related to the balance of forces between Stokes drag, the PRF and the SRF.

#### 3.2.3. Washing of Captured Nanoparticles

To better understand the dynamics that inhere in the interaction between plastic nanoparticles and silica clusters, we added a washing step after enrichment. This washing step allowed us to evaluate nanoplastic release under a constant, yet significant, flow rate of 0.5 mL/min, relieving any concerns about fluorescence being solely due to the flowing solution or background reflection.

**Figure 6:**
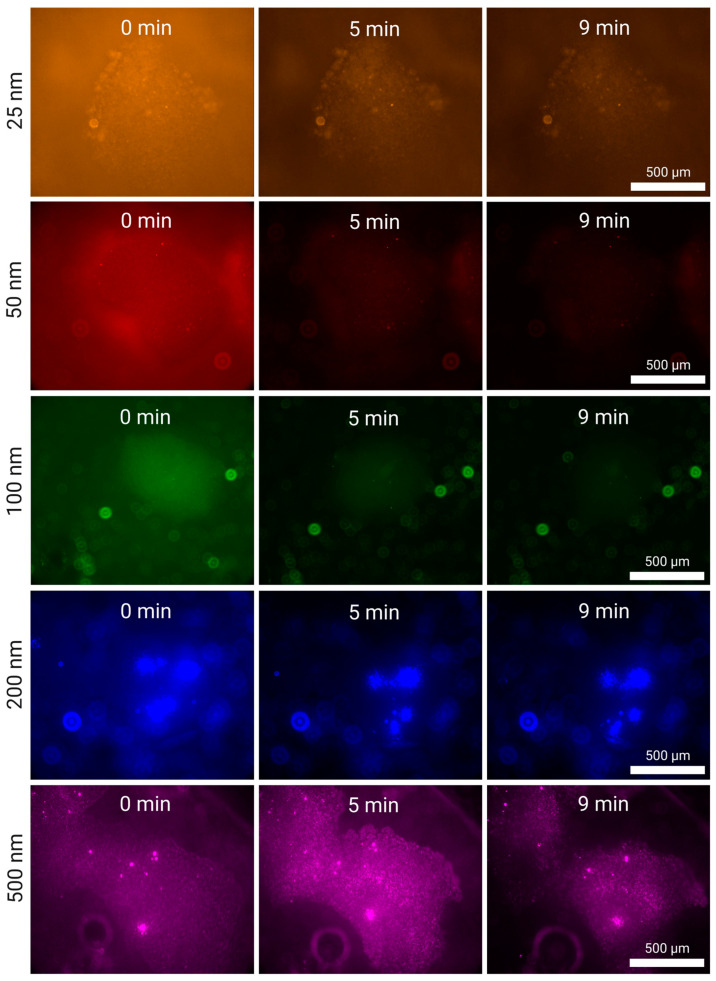
Nanoparticle washing from a silica cluster at a flow rate of 0.5 mL/min directly after enrichment at 5 mL/min. The decrease in fluorescence is attributed to the nanoparticles being washed away from the cluster. The particles used were 25 nm (red), 50 nm (orange), 100 nm (green), 200 nm (blue) and 500 nm (magenta).

**Figure 7:**
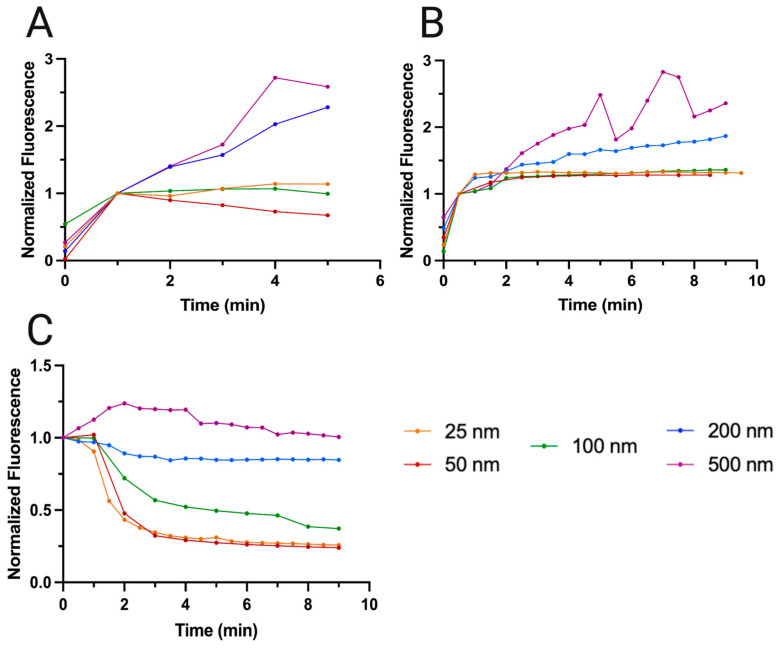
(**A**) Enrichment performance at 5 mL/min, for 4 min, for a total of 20 mL of solution processed. (**B**) Enrichment performance at 2 mL/min, for 10 min, for a total of 20 mL of solution processed. (**C**) Washing at 0.5 mL/min after the nanoparticle enrichment experiments. The decrease in fluorescence is attributed the nanoparticles being displaced by the flowing MQ water from their clusters. The particles used were 50 nm (red), 100 nm (green), 200 (blue) and 500 (magenta). The experiments were performed in duplicate, and plotted with their average values.

In Figure 6, we observe the tile set representing all the fluorescence images pertaining to these experiments after the 5 mL/min experiments (for the 2 mL/min experiments, refer to Figure S4), and in Figure 7C are graphed the values of these images, normalized to the first value.

In terms of washing (Figure 7C), we observe a general tendency for nanoparticles to wash away faster in the first quarter of the experiment (P1—t = 0 min to t = 2 min) compared to the rest of it (t = 2 to t = 9 min), with 500 nm as the only exception.

We observe (Table 1) that the fluorescence for 25 nm particles decays 56.7% within the first two minutes and only 16.9% further in the next six minutes. For 50 nm, these values are 52.4% in the next two minutes and a further 23.0% in the final six minutes. The smallest particles exhibit the sharpest decline in their fluorescence during washing, serving as a lower limit of what can be expected as the nanoparticles escape from the silica cluster.

The intermediate nanoparticle, 100 nm, decays 28.0% in the first two minutes and a further 33.5% by the eighth. It is more resistant to washing, as is expected. Its larger size invites a stronger contribution by the PRF, compared to the smaller nanoparticles.

The larger particles show the least fluorescence loss. For 200 nm, there is a loss of 10.9% in the first two minutes and a further 4.2% loss in the remaining six minutes.

The 500 nm particles show a different fluorescence loss curve altogether, with the analyzed clusters increasing in fluorescence above baseline. In the first two minutes, the cluster gains 23.8% of fluorescence signal. In the next six minutes, this fluorescence is lost until it is 2.8% below the baseline value at t = 0 min. This is because of the stronger influence of the PRF on the 500 nm particles when compared to others; specifically, as 500 nm particles are washed, they are immediately captured by the next cluster with greater ease, thereby increasing its fluorescence. After the excess nanoparticles have been washed from the clusters at large, the fluorescence begins decreasing below baseline.

By comparing these five nanoparticles, and their behavior in the silica grid, we can observe a size dependence in how they are released from the clusters. In general, we see a sharp decline in fluorescence at the beginning of each experience, with the exception of 500 nm. This decline is sharper for the smallest nanoparticles (25 and 50 nm) and is dampened as the particle grows larger and more sensitive to the size-scaling PRF. As size increases, the total fluorescence loss becomes smaller, until 500 nm becomes large enough to be so actuated by the PRF that its curve cannot be related to the other particles.

## 4. Conclusions

In this paper, we present the EchoTilt method for capturing nanoparticles at high flow rates. Building on our previous EchoGrid work, in the EchoTilt, we have developed an algorithm to simulate flow and particle interaction in the acoustic grid and generated a statistical score to select the best tilting angle to maximize capture. We applied the tilted acoustic field to our microfluidic channel, successfully using the S-ESP method to enrich nanoparticles in silica clusters from 500 nm down to 25 nm, even at a high flow rate of 5 mL/min, measuring this enrichment using fluorescence imaging. In addition, we evaluated, through a washing step with a buffer solution at 0.5 mL/min, the fluorescence decay of enriched clusters, showing the effect of particle size on release rates. We also showed how large nanoparticles are more susceptible to the PRF compared to the smaller nanoparticles, underlining the SRF’s impact in a system using the seed particle method for nanoparticle enrichment at the lower size ranges. In conclusion, the EchoTilt method provides a platform for studying the interaction between silica seed particles and nanoplastics even down to 25 nm, offering a new technique to upconcentrate and enrich these difficult-to-manage contaminants for endpoint analysis and monitoring.

## Figures and Tables

**Table 1:** Percentage of fluorescence lost relative to the first data-normalized data point during 10 min of washing at 0.5 mL/min. Negative values correspond to an increase in fluorescence compared to the first point.

	Fluorescence Loss per Particle Size (nm)				
Time (min)	25	50	100	200	500
2	56.7%	52.4%	28.0%	10.9%	−23.8%
4	69.1%	70.9%	47.9%	14.4%	−19.4%
6	72.3%	73.8%	52.3%	15.2%	−7.2%
8	73.6%	75.4%	61.5%	15.1%	−2.8%

## Data Availability

The original contributions presented in this study are included in the article/Supplementary Material. Further inquiries can be directed to the corresponding author.

## Supplementary Materials

The following supporting information can be downloaded at https://www.mdpi.com/article/10.3390/mi15121487/s1, Figure S1: COMSOL Multiphysics simulation results. (A) Simulated 7 × 7 grid after the eigenfrequency sweep, with highlighted yellow cutline from which acoustic pressure amplitude was extracted. (B) Graph of the total acoustic pressure along the grid nodes, which was then normalized to a 0–1 range for the Python algorithm. Figure S2: Silica-enhanced seed particle method large nanoparticle enrichment at a flow rate of 2 mL/min using the EchoTilt device. The increase in fluorescence is attributed to the flowing nanoparticle solution interacting with the cluster. The particles used were 100 nm (green), 200 nm (blue) and 500 nm (magenta). Figure S3: Silica-enhanced seed particle method small nanoparticle enrichment at a flow rate of 2 mL/min using the EchoTilt device. The increase in fluorescence is attributed to the flowing nanoparticle solution interacting with the cluster. The particles used were 25 nm (orange) and 50 nm (red). Figure S4: Nanoparticle washing from a silica cluster at a flow rate of 0.5 mL/min directly after enrichment at 2 mL/min. The decrease in fluorescence is attributed to the nanoparticles being washed away from the cluster. The particles used were 25 nm (red), 50 nm (orange), 100 nm (green), 200 nm (blue) and 500 nm (magenta). Table S1: Parameters used for COMSOL Multiphysics Pz-26 substrate simulation.
