# Supplementary material for: EchoTilt: An Acoustofluidic Method for the Capture and Enrichment of Nanoplastics Directed Toward Drinking Water Monitoring
Source: Micromachines (Basel). 2024 Dec 11;15(12):1487. doi: 10.3390/mi15121487 (PMC11728305; doi:10.3390/mi15121487)
Supplement: Supplementary file 1 [file micromachines-15-01487-s001.zip › micromachines-3337834-supplementary.pdf]

micromachines

EchoTilt: An Acoustofluidic Method for the Capture and Enrichment of Nanoplastics Directed Toward Drinking Water Monitoring

Martim Costa [1], Liselotte van der Geer [1], Miguel Joaquim [2], B. Hammarström [3], S. Tanriverdi [1], H. N. Joensson [1], M. Wiklund [3] and A. Russom [1,4,*]

1 Science for Life Laboratory, Department of Protein Science, Division of Nanobiotechnology, KTH Royal Institute of Technology, 171 65 Solna, Sweden; martimc@kth.se (M.C.); lgbvdg@kth.se (L.v.d.G.); selimt@kth.se (S.T.); hakan.jonsson@scilifelab.se (H.N.J.)
2 Departamento de Bioengenharia, Instituto Superior Técnico, 1049-001 Lisboa, Portugal; miguelrtsjoaquim@gmail.com
3 Science for Life Laboratory, Department of Applied Physics, KTH Royal Institute of Technology, 171 65 Solna, Sweden; bham@kth.se (B.H.); martin.wiklund@biox.kth.se (M.W.)
4 AIMES Center for the Advancement of Integrated Medical and Engineering Sciences at Karolinska Institutet and KTH Royal Institute of Technology, 171 65 Solna, Sweden
* Correspondence: aman.russom@scilifelab.se

Supplementary Information

GitHub Link for Python Code:
https://github.com/miguel436/grid_simulation/tree/main

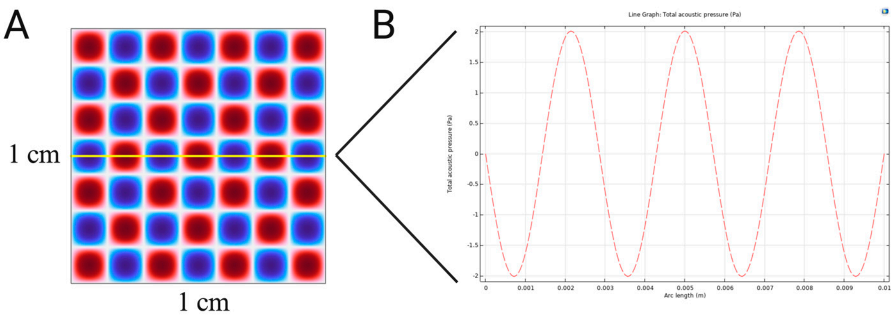

Figure S1. COMSOL Multiphysics simulation results. (A) Simulated 7×7 grid after the eigenfrequency sweep, with highlighted yellow cutline from which acoustic pressure amplitude was extracted. (B) Graph of the total acoustic pressure along the grid nodes, which was then normalized to a 0-1 range for the Python algorithm.

Geometry Materials	*Physics Module*	*Mesh*	*Study*	*Results and Datasets*
Square Substrate 1 cm side.<br><br>***Pz-26 Shear***<br><br>***Density***<br><br>*7700 kg/m*^{3}<br><br>***Speed of sound***<br><br>*3600 m/s*	***Pressure Acoustics, Frequency Domain***<br><br>***Out-of-plane wave number:***<br><br>*0*<br><br>***No port sweep***<br><br>***Amplitude normalization***<br><br>***Use reference pressure for air***<br><br>c_{ref} *= 343 m/s*<br><br>***Sub-modules: Pressure Acoustics***<br><br>***Sound Hard Boundary (Wall)***<br><br>***Initial Values = 0***<br><br>***Sound Soft Boundary.***	***User-controlled mesh.***<br><br>*Calibrated for General physics.*<br><br>*Extra fine element size.*<br><br>*Free Triangular*<br><br>Vertices/Triangles 3242/6282<br>Edge/Vertex 200/4<br><br>***Element Statistics***<br>*Number: 6282*<br>*Minimum element quality: 0.6911*<br>*Average element quality: 0.9541*<br>*Element area ratio: 0.353*<br>*Mesh area:*<br>*1.0E-4 m*^{2}<br>*Element Quality Histogram:*<br>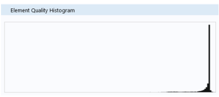	***Eigenfrequency***<br><br>***ARPACK Solver***<br><br>***Search method:*** *Around shift*<br><br>***Desired number of eigenfrequencies:*** *20*<br><br>***Unit:*** *Hz*<br><br>***Eigenfrequencies around:*** *1.8 MHz*<br><br>***Search method around shift:*** *Closest in absolute value*	*Cut line function*<br><br>*1D Plot Group*

Table S1. Parameters used for COMSOL Multiphysics Pz-26 substrate simulation.

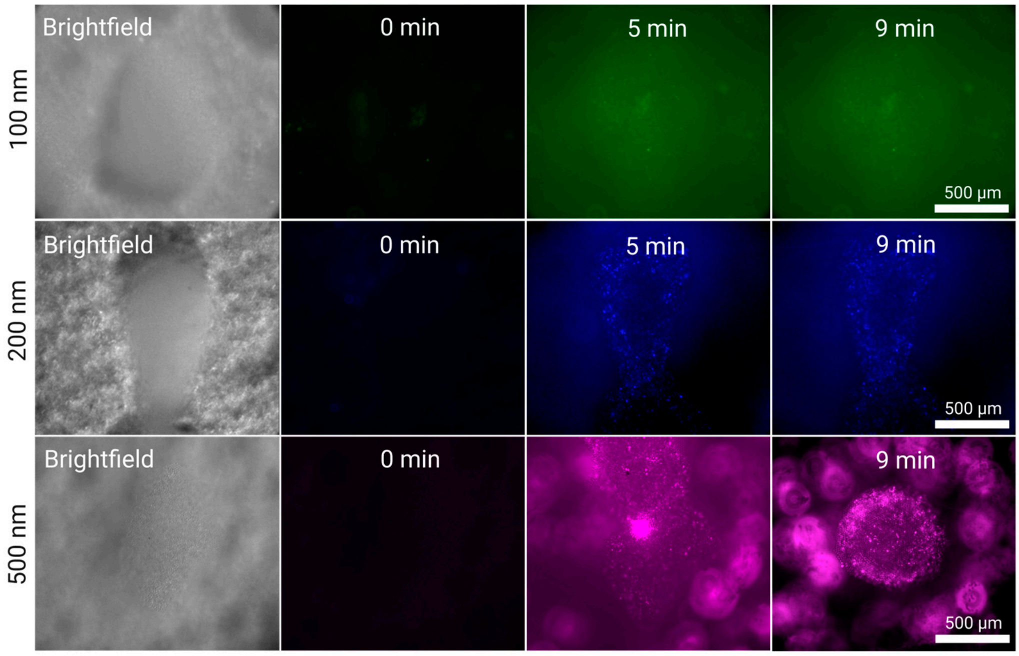

Figure S2. Silica-Enhanced Seed Particle Method large nanoparticle enrichment at a flow rate of 2 mL/min using the EchoTilt device. The increase in fluorescence is attributed to the flowing nanoparticle solution interacting with the cluster. The particles used were 100 nm (green), 200 nm (blue) and 500 nm (magenta).

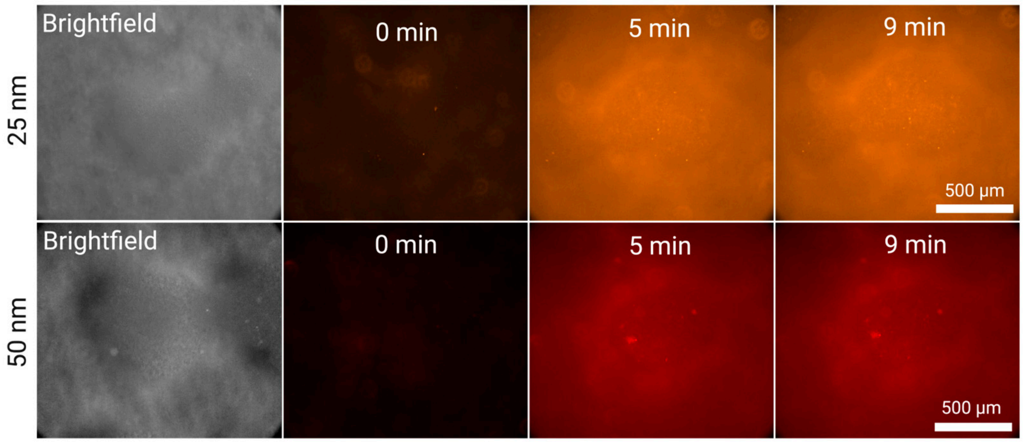

Figure S3. Silica-Enhanced Seed Particle Method small nanoparticle enrichment at a flow rate of 2 mL/min using the EchoTilt device. The increase in fluorescence is attributed to the flowing nanoparticle solution interacting with the cluster. The particles used were 25 nm (orange) and 50 nm (red).

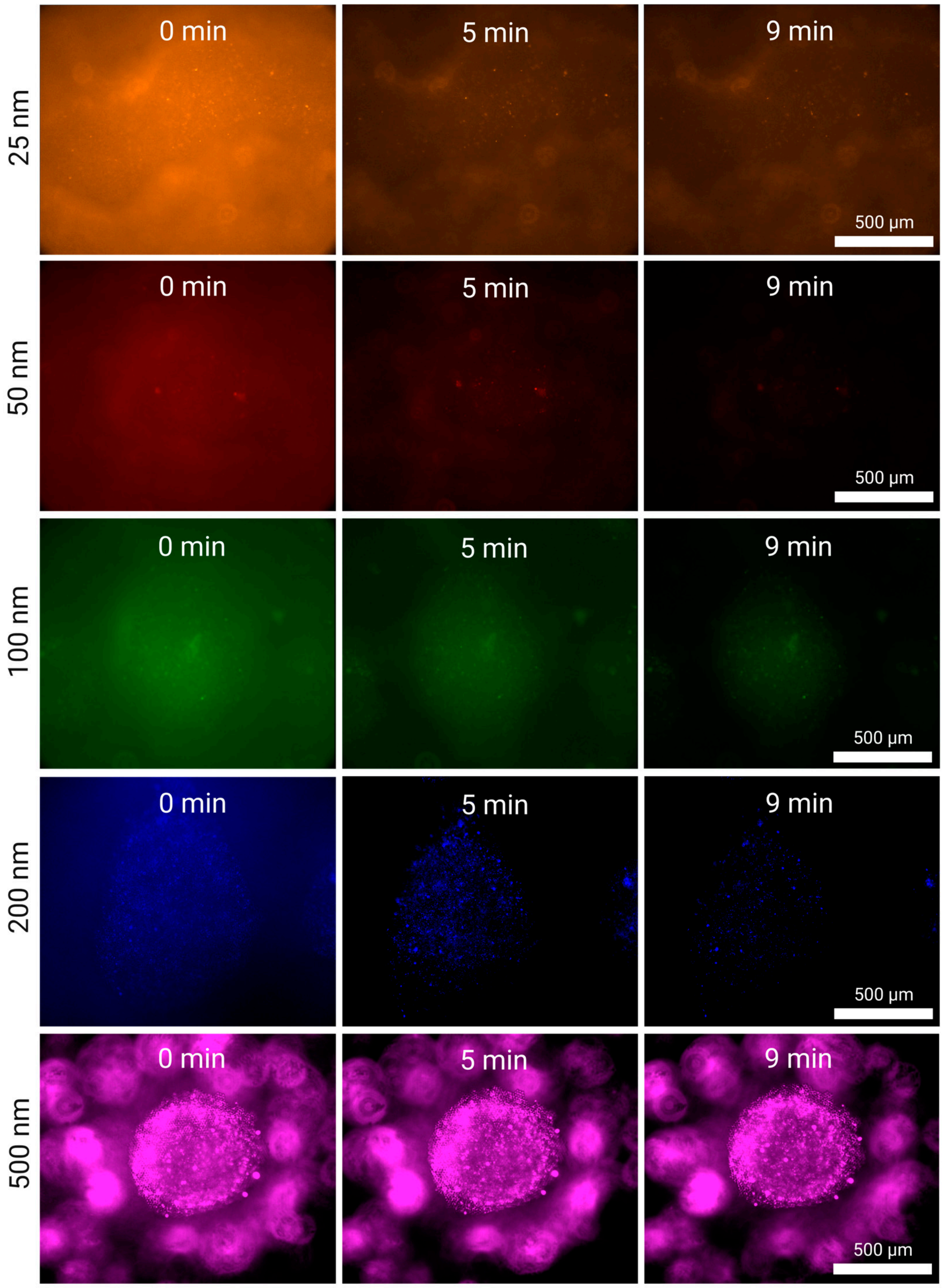

Figure S4. Nanoparticle washing from a silica cluster at a flow rate of 0.5 mL/min directly after enrichment at 2 mL/min. The decrease in fluorescence is attributed to the nanoparticles being washed away from the cluster. The particles used were 25 nm (red), 50 nm (orange), 100 nm (green), 200 nm (blue) and 500 nm (magenta).
